# Deep neural network for the determination of transformed foci in Bhas 42 cell transformation assay

**DOI:** 10.1038/s41598-021-02774-2

**Published:** 2021-12-02

**Authors:** Minami Masumoto, Ittetsu Fukuda, Suguru Furihata, Takahiro Arai, Tatsuto Kageyama, Kiyomi Ohmori, Shinichi Shirakawa, Junji Fukuda

**Affiliations:** 1 Faculty of Engineering, Yokohama National University, 79-5 Tokiwadai, Hodogaya-ku, Yokohama, Kanagawa 240-8501 Japan; 2 Graduate School of Environment and Information Sciences, Yokohama National University, 79-7 Tokiwadai, Hodogaya-ku, Yokohama, Kanagawa 240-8501 Japan; 3 Kanagawa Institute of Industrial Science and Technology, 3-2-1 Sakado Takatsu-ku, Kawasaki, Kanagawa 213-0012 Japan; 4 Kanagawa Prefectural Institute of Public Health, 1-3-1 Shimomachiya, Chigasaki, Kanagawa 253-0087 Japan

**Keywords:** Chemical safety, Cancer models, Image processing

## Abstract

Bhas 42 cell transformation assay (CTA) has been used to estimate the carcinogenic potential of chemicals by exposing Bhas 42 cells to carcinogenic stimuli to form colonies, referred to as transformed foci, on the confluent monolayer. Transformed foci are classified and quantified by trained experts using morphological criteria. Although the assay has been certified by international validation studies and issued as a guidance document by OECD, this classification process is laborious, time consuming, and subjective. We propose using deep neural network to classify foci more rapidly and objectively. To obtain datasets, Bhas 42 CTA was conducted with a potent tumor promotor, 12-O-tetradecanoylphorbol-13-acetate, and focus images were classified by experts (1405 images in total). The labeled focus images were augmented with random image processing and used to train a convolutional neural network (CNN). The trained CNN exhibited an area under the curve score of 0.95 on a test dataset significantly outperforming conventional classifiers by beginners of focus judgment. The generalization performance of unknown chemicals was assessed by applying CNN to other tumor promotors exhibiting an area under the curve score of 0.87. The CNN-based approach could support the assay for carcinogenicity as a fundamental tool in focus scoring.

## Introduction

Assessing the carcinogenic risk of over-the-counter chemicals and ever-increasing new chemicals is an important issue globally.^1^ Because DNA damage and mutations are thought to trigger carcinogenesis, short-term in vitro and in vivo genotoxicity tests have been used as a method to predict the carcinogenicity of chemical substances. However, a large number of laboratory animals, time, and cost are required to meet all the regulations of the current carcinogenic risk assessment.^2,3^ Furthermore, not all carcinogens are genotoxic.^4^ Improving the reliability and predictability of in vitro cell transformation assay (CTA), including the detection of nongenotoxicity, will lead to the realization of 3R principles for animal experimentation in chemical safety testing.^5,6^

An In vitro CTA uses the induction of phenotypic changes that occur in cells having tumorigenic potential in vivo as an index.^7^ Cells with phenotypic characteristics of malignant cells form tumors in susceptible animals.^8^ This supports the use of specific phenotypic changes in vitro as an index for predicting carcinogenicity in vivo.

In vivo carcinogenesis studies have also shown that carcinogenesis is a multistage process comprising clearly distinguishable initiation, promotion, and progression.^9,10^ In vitro CTA can partially imitate initiation and promotion.^11,12^ In general, many genotoxic carcinogens cause initiation, and many nongenotoxic carcinogens cause promotion.^13^ Some of the in vitro assays published in the OCED guidelines detect initiator with genotoxicity as an index. Bhas 42 CTA is to predict carcinogenesis of compounds, which is the only method published in the OECD guidance document that can distinguish tumor promoters and initiators.^14,15^.

Bhas 42 cells were established by introducing the *v-Ha-ras gene* into Balb/3T3 A31-1–1 cells.^16^ These cells form a contact-inhibited confluent monolayer, but when exposed to carcinogens, the transformed cells proliferate to form transformed foci. The Bhas 42 CTA evaluates the potential of chemicals to cause initiation or promotion using the frequency of focus occurrence as an index.^17^ In the promotion test, the exposure of cells to chemicals is carried out at a higher cell density than the initiation test. Subcutaneous implantation of untransformed Bhas 42 cells into a nude mouse did not form a tumor, but transformed focus cells showed 100% tumorigenicity (four out of four).^18^ Further, the expression of the *v-Ha-ras gene* is 2 to 14 times higher in focus cells than in untransformed cells.^15^ In addition to the multilaboratory collaborative study of Bhas 42 CTA, an international validation study of the Bhas 42 CTA has been conducted by the Japanese Center for the Validation of Alternative Methods (JaCVAM).^19,20^ EURL ECVAM reviewed the studies and recommended Bhas 42 CTA based on its transferability, reproducibility and relevance of the protocol.^21^ However, EURL ECVAM also points out that sufficient education and training for determiners are essential because focus scoring is done visually.^15^ A photo catalog of various examples of untransformed and transformed foci are provided to assist in distinguishing transformed foci and nontransformed cell clusters. Nevertheless, the scoring task is labor intensive, time-consuming, and inevitably subjective because various morphological foci are observed.

The purpose of this study is to develop and implement an automatic assessment tool using deep learning to support focus scoring in Bhas 42 CTA. The focus images of the Bhas 42 CTA are used to train a convolutional neural network (CNN) used for image recognition and to evaluate the classification performance of the focus images. The advantage of a CNN is that it does not require explicit feature extraction and can learn the feature extraction from data. Moreover, there is a need to manually design a feature extraction method when using machine learning models such as linear models and decision trees. In addition, CNNs have achieved great success in the field of computer vision, including biomedical image recognition.^22,23^ Therefore, a CNN was selected to determine the transformed foci in this study. We also evaluated whether CNN could determine the focus induced by promoter compounds that were not used during training. In addition, the CNN judgment was compared with the conventional classifiers by beginners learned using the OECD guidance document. This CNN automatic determination may be useful in scoring cell assays such as Bhas 42 CTA that use focus formation and cell morphological changes as indexes.

## Results and discussion

### Bhas 42 CTA

**Figure 1:**
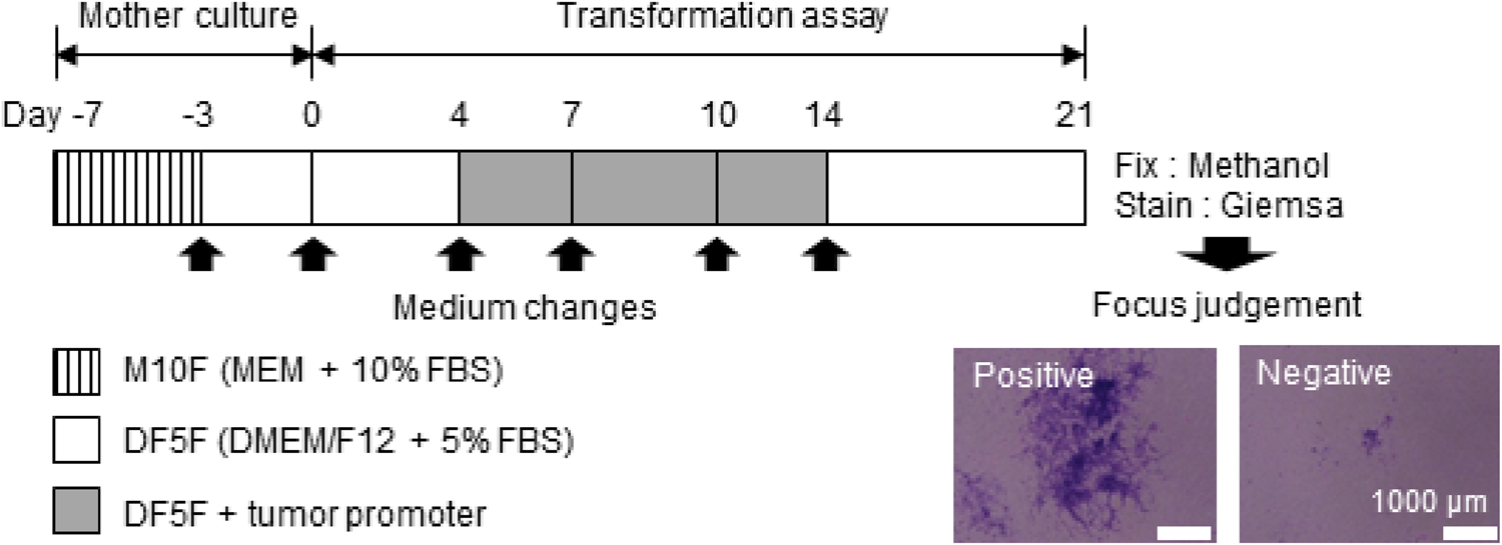
Experimental procedures of Bhas 42 cell transforming assay (Bhas 42 CTA). Bhas 42 cells were precultured in M10F medium for 4 days and in DF5F medium for 3 days. The cells were then seeded in a 6-well plate (culture, 0 day). On the 4th day of culture, the test chemical was added to DF5F medium and exposed for 10 days. These cells were cultured in plain DF5F medium for another 7 days. Giemsa staining was performed on the 21st day of culture, and micrographs of areas with potential transformed foci were taken. Two experts performed both positive and negative judgments.

As shown in Fig. 1, the Bhas 42 CTA was conducted to acquire data according to the OECD guidance document. Briefly, Bhas 42 cells were precultured for 7 d and the cells were harvested. The cells were seeded on a 6-well plate, cultured for 4 d and then exposed to 12-*O*-Tetradecanoyl-phorbol-13-acetat (TPA, 0, 5, 10, 20, 50 ng/ml) for 10 d. The cells were subsequently stained with Giemsa after culturing for 7 d in plain medium. Regions of cell aggregates with the potential of transformed focus were photographed. The positive/negative judgment of focus was done by two experts based on six criteria (basophilic, spindle shape, multilayer, random, invasive, and 100 or more cells forming focus).^15^ If the judgment was divided between the two experts, it was decided after discussion. Typical positive and negative cell images are shown in Fig. 1. The total number of images was 1405. The breakdown is shown in Table 1. In Bhas 42 CTA, it is deemed that promotion activity occurs for a chemical when the frequency of cell transformation is increased statistically significantly at two consecutive set concentrations. In this experiment, the number of foci counted by the experts was 0.16 ± 0.41 at a TPA concentration of 0 ng/ml, whereas it was 2.17 ± 2.04 at 10 ng/ml, 2.17 ± 1.47 at 20 ng/ml, and 2.67 ± 1.86 at 50 ng/ml. One-way analysis of variance and Dunnett t-test observed a statistically significant difference (*P* < 0.05) at 10 and 20 ng/ml for a TPA concentration of 0 ng/ml, and TPA was correctly judged as a promoter.

**Table 1:** Number of images of suspected foci.

Chemical	Positive	Negative
TPA	1087	297
DMSO	15	6
Total	1102	303

### Training and performance of CNN

**Figure 2:**
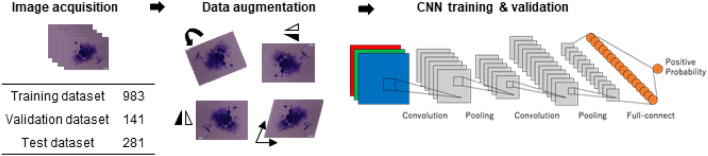
Deep learning with datasets on Bhas CTA. A total of 1405 datasets were acquired by Bhas 42 CTA and classified into 3 data: 983 training data (70%), 141 validation data (10%), and 281 test data (20%). During the training, data augmentation such as rotation and translation was applied to the input images, and data augmentation was performed. CNN training was carried out for 50 epochs, and the parameters of CNN when the accuracy for validation data became maximum were used. Trained CNNs were evaluated with test data.

**Figure 3:**
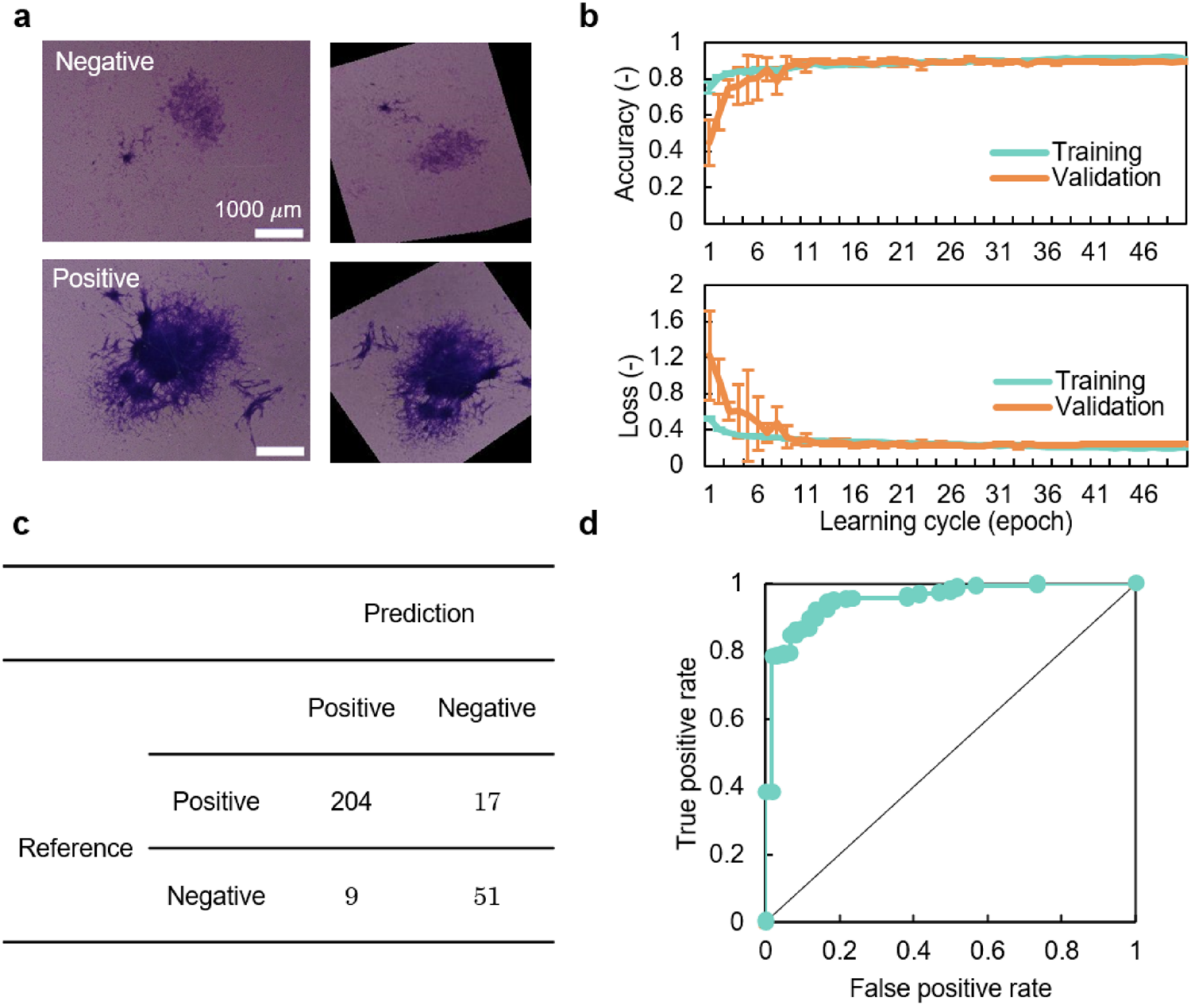
Performance of CNN algorithm. (**a**) Representative images of transformed foci induced by exposure to TPA. Two experts classified the images into positive and negative foci. (**b**) Learning curves. The mean and standard deviation for 5 independent trials are plotted. (**c**) Confusion matrix. The confusion matrix when the AUC value is the median value out of 5 trials is posted. (**d**) ROC curve for test dataset. ROC curve when AUC value is median value out of 5 trials. The average AUC of 5 trials is 0.95 (± 0.008).

CNN is a neural network model used for image recognition tasks in deep learning, and has been applied to various fields including medicine and biology.^22–25^ Here, as shown in Fig. 2, the images of transformed focus candidates obtained by Bhas 42 CTA were randomly divided into training data (70%), validation data (10%), and test data (20%). For CNN, 18-layer ResNet^26^ was used. The input images were resized to 224 × 224 and input to CNN, and data augmentation processing such as rotation and translation was applied during training. Training of CNN was conducted for 50 epochs using training data, and performance against test data was evaluated using CNN when classification accuracy for validation data became maximal. Due to the randomness of CNN training, five independent trials were conducted. Figure 3a shows an example of image data that has undergone data augmentation. Figure 3b is a learning curve. The accuracy rate increased with increase in epoch, and converged at about 0.89. The loss value decreased with increase in epoch, and converged to about 0.24. The accuracy rate/loss value of the training data set and the validation data set converge to almost the same value, suggesting that CNN training is possible without causing overfitting. Figure 3c shows the confusion matrix of test data. When the average and standard deviation were calculated from the accuracy rate and recall rate of the five trials, the accuracy rate was 0.89 ± 0.012 and the recall rate was 0.91 ± 0.015. The fine-tuning technique using the pre-trained model on a large dataset such as ImageNet is a standard approach when the dataset size is limited. Although we used the ImageNet pre-trained model implemented in PyTorch in the preliminary experiment, the effect was limited. Therefore, it was not used for CNN training in this study. Figure 3d shows the receiver operating characteristic (ROC) curve when the area under the curve (AUC) value became the median among the five trials. The AUC value for the 5 trials was 0.95 ± 0.008. The AUC value is 0.5 for a completely random model and 1.0 for the ideal, and this value suggests that this CNN has excellent performance. To compare the capability of the CNN model with non-CNN approaches, we built a focus prediction model using three hand-crafted features and logistic regression based on a previous study that attempted to classify the focus images in Balb/c 3T3 CTA.^27^ Note that we used the same dataset as the one used for the CNN model. The accuracy, recall, and AUC values were 0.82, 0.94, and 0.86, respectively. These results suggest that the CNN model is superior to the model proposed in the previous study in terms of accuracy and AUC value.

### Comparisons with conventional classifier

**Figure 4:**
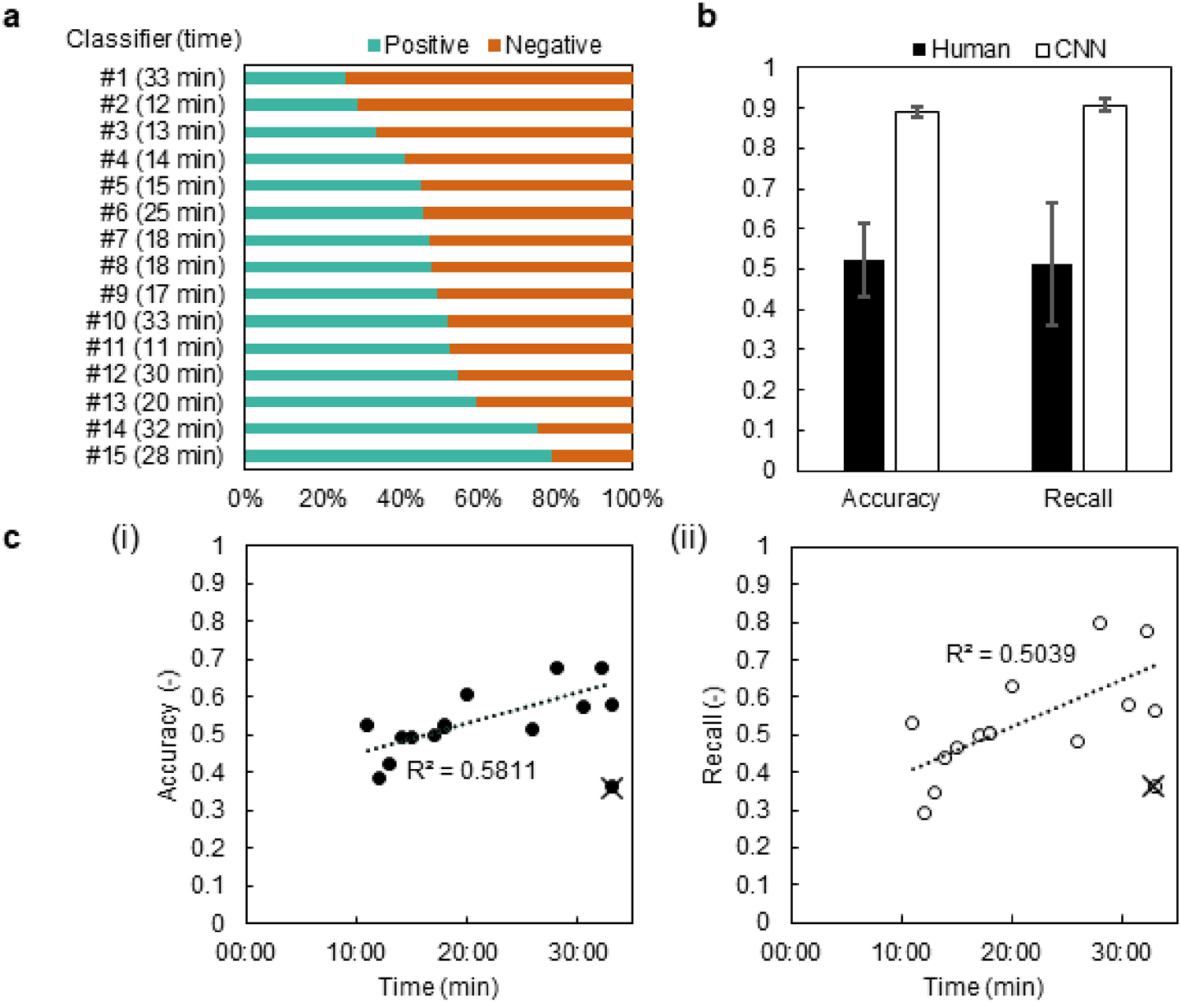
Comparisons of conventional classifier and CNN-based algorithm. (**a**) Positive and negative discrimination by beginner classifiers. Fifteen people classified 281 focus images. The time values indicate time period required to classify all the images. (**b**) Comparisons of conventional and CNN-based classification. Conventional classification values indicate the mean and deviation of 15 measurers. CNN-based values indicate the mean and deviation of 5 trials. (**c**) The time required for the judgment and the accuracy rate (i), and the reproducibility (ii) are excluded from the calculation of the regression line by the least squares method as outliers.

Fifteen volunteers read the evaluation criteria and image atlas mentioned in the OECD guidance document and judged 281 test data. They are beginners with experience in cell culture but no experience in Bhas 42 CTA. We designed the experiments considering situations in which the assay was initially tested to estimate its applicability. This could be an essential step for cell-based assays, especially for public test methods, to gain wider use worldwide. During the initial feasibility testing, we assumed that operators with experience in cell culture but no experience in Bhas 42 CTA at cosmetics and pharmaceutical companies or contract research organizations classify the transformed foci by referring to the six evaluation criteria and image atlas of the OECD guidance document. Figure 4a shows the judgment result and required time of each beginner classifier. The percentage judged as positive was widely distributed in the range of 20–80%. As shown in Fig. 4b, the accuracy rate and recall rate of beginner classifiers were shown to be considerably lower than those of the CNN. Also, the time required for the judgment was 21.3 min on average (maximum 33 min, minimum 11 min). As shown in Fig. 4c, there is a slight positive correlation between the required time and the accuracy rate/recall rate. In addition, as shown by the outliers in Fig. 4c, it was found that the accuracy and recall rates were low even over time, and there are people who are not suitable for such image judgment. Although the data may provide valuable insights into comparing CNN and conventional classifiers, we have to note that they were obtained from a limited number of volunteers.

In Bhas 42 CTA, it is judged that a chemical possesses promotion activity when the number of focus under two conditions of higher concentration is significantly larger than the number of focus in a certain concentration. That is, it is not the absolute number of focus, but a prediction by the relative number of focus at a given concentration in the experiment. Therefore, if the same individual performs the judgment of focus at all concentrations, it is said that there is no problem even if there is a difference in the recognition of the focus between individuals. In previous reports, the interlaboratory reproducibility of Bhas 42 CTA was good, with a concordance rate between 3 facilities of 83% (10/12) among the 12 substances used in the test.^19^ However, to prevent data fluctuations due to subjective judgment, appropriate training of determiners by CTA-experienced persons inside and outside the facility and second opinions (referring to the opinions of other decision makers or experienced decision makers) should be sought. The data presented here suggest that it is difficult to eliminate subjectivity in conventional classification and that sufficient training is required. CNN may be able to support more objective implementation of judgment in various in vitro cell based assays, including Bhas 42 CTA.

### Application of CNN to other tumor promotion chemicals

Because TPA is a typical promoter, we have so far used it to train CNN and tested whether the same compound can be detected. However, the purpose of CTA is to predict carcinogens among compounds of unknown carcinogenicity. Therefore, we selected lithocholic acid (LCA) and 1-nitropyrene (1-NP) from a list of compounds considered as promoters, and evaluated whether they could be detected using CNN trained using images of TPA exposure. It should be noted that focus images exposed to LCA and 1-NP are not used for CNN training.

**Figure 5:**
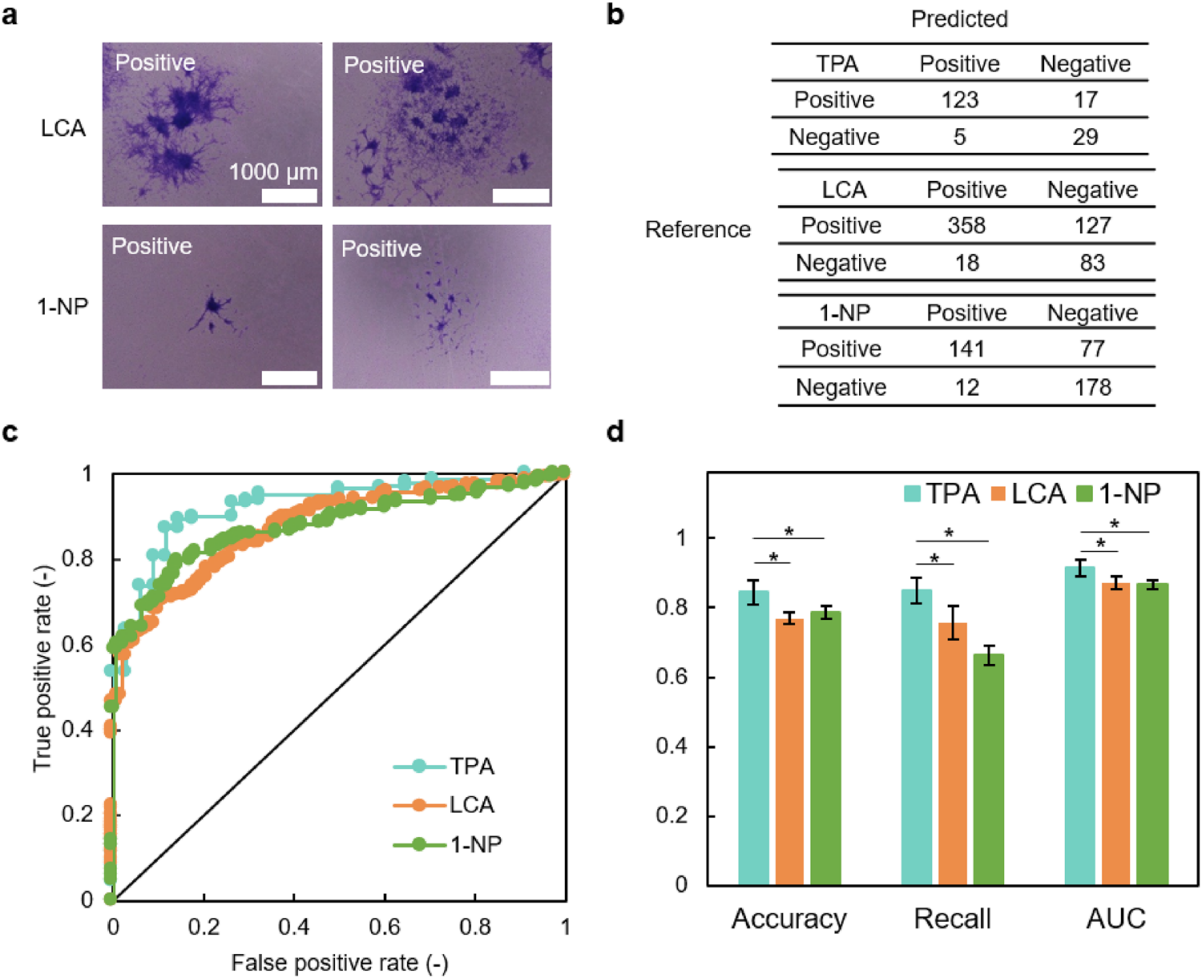
Application of CNN algorithm to other promoters. (**a**) Representative images of transformed foci induced by exposure to LCA and 1-NP. (**b**) Confusion matrix. The Confusion matrix when the AUC value is the median value out of 5 trials is posted. (**c**) ROC curves. The ROC curve of the trial when the AUC value became the median value out of 5 trials is posted. (**d**) Comparisons of CNN performance for three chemicals. The values indicate the mean and deviation of 5 trials. Statistical significant difference was assessed by one-way analysis of variance and Dunnett's *t* test. *P < 0.01 was considered significant.

Figure 5a shows a typical LCA, 1-NP focus. LCA formed a clear focus, which was mainly large and dark. 1-NP formed a smaller microfocus. Such a focus is also seen in TPA, but the proportion is not high. A dataset of positive and negative judgments by two experts was prepared. Table 2 shows the number of captured images. As a positive control, the TPA exposure experiment was also conducted at the same date and time and using the same procedure. When learning CNN, the focus candidate images shown in Table 1 were randomly divided into training (90%) and validation (10%). The experimental settings for CNN are the same as those for the previous experiment. In addition to the LCA and 1-NP focus candidate images shown in Table 2, the TPA focus candidate images for positive control were also used as the test images.

**Table 2:** Number of images of suspected foci in different chemicals.

Chemical	Positive	Negative
TPA	140	34
LCA	485	101
1-NP	218	190
DMSO	18	5
Total	861	330

Figures 5b,c show the confusion matrix and ROC curve, respectively. Further, Fig. 5d shows the average value of accuracy rate, recall rate, and AUC for the five trials. The AUC value (0.91 ± 0.025) at TPA exposure was lower than the value (0.95 ± 0.008) shown in Fig. 3d. The difference in these AUC values is likely due to the data splitting method. In Fig. 3, the test data are sampled from the data source shown in Table 1. Therefore, the data used in training and test phases are considered to have a similar trend, resulting in the high AUC value of 0.95. On the other hand, the test data used for Fig. 5d come from the dataset shown in Table 2 and are different data groups from the dataset used to train the CNN model. We consider this dataset difference causes the difference in the AUC values. Additionally, as shown in Fig. 5d, the accuracy, recall, and AUC of LCA and 1-NP were lowered compared to TPA. The AUC values for LCA and 1-NP were 0.87 ± 0.013 and 0.87 ± 0.018, respectively. When applying the logistic regression model with three hand-crafted features in the same scenario, the AUC values for TPA, LCA, and 1-NP were 0.88, 0.87, and 0.84, respectively. The AUC values for TPA and 1-NP given by the CNN model were higher than those from logistic regression, whereas the AUC value for LCA was the same in both models. Therefore, our CNN model is still advantageous compared to the previous approach when applying the trained model to different data groups and other compounds. The experimental results indicates that CNNs trained using one compound reduced the detection performance of the other compounds. In particular, the judgment accuracy was reduced in compounds that induce morphologically different characteristics (microfocus) such as 1-NP. More than 45 compounds, including TPA, LCA, and 1-NP, were considered tumor promoters through in vivo testing and Bhas 42 CTA.^14,28^ Additional training on CNNs using these compounds will enable the detection of potential unknown promoters. Further, the code of the CNN used this study is revealed to the public (Supplementary data). This also allows images with different setups to be collected, such as experiment dates, experimenters, and experimental facilities, to investigate the generality and robustness of this CNN in public. The Bhas 42 CTA has been designed as an end-point assessment, taking into account human judgment. However, it may be possible to make a more accurate judgment by CNN by using a large amount of data such as time course of changes in cell morphology, migration, and proliferation. We monitored changes in cell morphology over time without staining and showed that cell differentiation function could be predicted from several cell morphology indicators.^29^ Recent advances in technology such as CNN and cell monitoring systems have the potential to innovate conventional cell-based toxicity testing.

In summary, this study suggested that the subjective, time consuming, and labor-intensive decision-making process in the focus determination of Bhas 42 CTA can be performed objectively and quickly using CNN. Using the same dataset for training and test phases, the AUC was found to reach 0.95. It was also shown that the performance of this CNN is considerably higher than the beginner classifiers who read the evaluation criteria and image atlas of the OECD guidance document to make judgment. However, it was shown that the use of different datasets for training and test phases degraded CNN performance, and that the detection of compounds that are not used in CNN learning further degrades performance. While it is clear that further training of CNN using other promoters and data sets of different culture dates, experimenters, facilities, etc., is required, the approach presented here may be a useful tool for transformation assays including Bhas 42 CTA.

## Limitations and future work

This study demonstrated that CNNs potentially support the implementation of the judgment of foci on Bhas 42 CTA more rapidly and objectively. However, this study still contained subjective biases and time-consuming steps. Our experimental verification was a single-case study. We only used the dataset collected by specific equipment and experimenters, implying that our dataset partially contains subjective biases from the experimenters. We cannot ensure that our CNN-based classifier works well under the other conditions. Another bias may be caused by experimenters taking microscopic images of regions of cell aggregates with the potential of transformed focus. This is laborious and time-consuming, which may limit and partially compromise our approach's rapid and objective classification. The use of electrically driven microscopy, an autofocus optical system, and a detection algorithm for potentially transformed foci to collect focus image data could be the subject of our subsequent study. Moreover, we evaluated only the CNN performance for limited tumor promotion chemicals. Evaluating the CNN-based classifier for other tumor promotion chemicals is an important future work to demonstrate the robustness and generality of our approach.

Introducing more sophisticated CNN algorithms may further enhance classification performance. For instance, several studies have revealed that ensemble architectures are effective in biomedical image-related classifications with noisy and small datasets.^30,31^ A well-known limitation of CNNs is the interpretability problem due to the black-box property. While a CNN is advantageous because it does not require explicit image feature extraction, hand-crafted feature-based machine learning, such as logistic regression, is superior in terms of interpretability. Statistical models, such as Bayesian models, are helpful when estimating a chemical to be carcinogenic or not after image-based determination of foci, because explanatory variables are given in such a phase. The Bayesian model developed by Stefanini and Magrini is targeted for Type III transformed focus in Balb c CTA.^32^ Five of the six criteria for the determination of Type III transformed focus in Balb c CTA are identical to those in Bhas 42 CTA, and the only difference is the number of cells that constitute the transformed focus. Therefore, if we can confirm the high performance of a CNN as a type III judgment model in Balb c CTA in the future, it is anticipated that the CNN will be integrated with the model developed by Stefanini and Magrini in Balb c CTA. Furthermore, several studies attempted to combine hand-crafted features and CNN features, which is a promising future direction to exploit the advantages of two different approaches.^33–36^ Although it is not easy to understand the CNN's classification mechanism, explanation techniques, such as Grad-CAM^37^ and LIME^38^, would be useful to gain some insights into the classification mechanism.

## Materials and methods

### Reagents

The reagents used for cell isolation, culture, and analysis were as follows: Bhas 42 cell from JCRB cell bank (Japan); phosphate-buffered saline (PBS) from Thermo Fisher Scientific (USA); 0.25w/v% Trypsin Solution with Phenol Red from FUJIFILM Wako Pure Chemical (Japan); Minimum essential medium (MEM) from Thermo Fisher Scientific (USA); Dulbecco’s Modified Eagle Medium/F12 (DMEM/F12) from Thermo Fisher Scientific (USA); Fetal bovine serum (FBS) from Moregate biotech (Australia); Giemsa's azur eosin methylene blue solution from Merck (USA); Petri Dish for Cell/Tissue Culture 90Φ (deep) from Sumitomo Bakelite (Japan); Multiwell Plate for Cell/Tissue Culture 6F with lid from Sumitomo Bakelite (Japan); Dimethyl sulfoxide for molecular biology (DMSO) from Sigma-Aldrich (USA); Methanol from FUJIFILM Wako Pure Chemical (Japan); PMA for use in molecular biology applications (TPA), ≥ 99% (HPLC) from Sigma-Aldrich (USA); Lithocholic acid (LCA) from Tokyo Chemical Industry (Japan); 1- Nitropyrene (1-NP) from Tokyo Chemical Industry (Japan).

### Preparation of dataset in Bhas 42 CTA

Bhas 42 cells were seeded in a 90 mm culture dish at a density of 5.0 × 10^4^ cells/dish and cultured in M10F (MEM + 10% FBS) medium for 4 d. Cells were exfoliated from the dish using 0.25% trypsin, suspended in DF5F (DMEM/F12 + 5% FBS medium), and seeded in a 90 mm culture dish at a density of 5.0 × 10^5^ cells/dish. After culturing for 3 days, the cells were exfoliated from the dish using 0.25% trypsin, suspended in DMEM/F12 + 5% FBS medium, and seeded on a 6-well plate at a density of 1.4 × 10^4^ cells/well. After culturing for 4 d, the culture medium was exchanged to DF5F containing TPA (50, 20, 10, 5 ng/ml), LCA (25, 20, 15, 10, 5 μg/ml), or 1-NP (1.5, 1.0, 0.8, 0.6, 0.4, 0.2 μg/ml), in which the cells were exposed for 10 d. The concentration of DMSO in any medium was set to 0.1%. One 6-well plate per concentration was used. Medium exchange with DF5F containing the test chemicals was performed on Day 7 and Day 11. It was replaced with plain DF5F on Day 14, and cultured for another 7 d. On Day 21 of culture, the cells were fixed with methanol and stained with Giemsa solution. The focus candidate regions where the cells are stacked were numbered and photographed using a digital microscope (KEYENCE, VHX-970F). The image was saved in jpeg format with a magnification of 1000 times, brightness of 1/120 s, and size of 2048 × 1536 or 1600 × 1200. Positive/negative judgment of focus was performed based on the six criteria (Basophilic, Spindle shape, Multilayer, Random, Invasive, focus with 100 or more cells) and image atlas described in the OECD guidance document^39^ by a focus determination expert or beginner.

### Creating a judgment model

We used a convolutional neural network (CNN) as the image classification model. A basic CNN architecture consists of convolution, pooling, and fully connected layers, and takes an image array as input. The convolution and pooling layers transform image-like array data using localized calculations. The roles of the convolution and pooling layers are to extract image features and spatially aggregate the extracted features. In the CNN, the weight parameters in each layer were optimized using training data. Implementation was conducted using Python 3.6.9, and ResNet18^26^ implemented in PyTorch 1.8.0^40^ was used for the CNN. In the TPA classification experiment, the transformed focus candidate images obtained by Bhas 42 CTA shown in Table 1 were randomly divided into training data (70%), validation data (10%), and test data (20%), and used in training and evaluation of CNN. Additionally, in the prediction for unknown substances, the focus candidate images shown in Table 1 were randomly divided into training (90%) and validation data (10%), and the CNN training was performed again, and the images shown in Table 2 were predicted and evaluated as test data. However, the ratio of Positive to Negative was approximately equal in each division. The input images were resized to 224 × 224 and input to CNN. During CNN training, random rotation in the range of [− 90°, 90°], random translation in the range of [− 44, 44] pixels, random shear in the range of [− 15°, 15°], and horizontal or vertical flipping with a probability of 0.5 were applied to input images as data augmentation. The binary cross entropy function was used for the loss function. The initial learning rate was set to 0.0001, and the learning rate was decayed using Cosine Annealing.^41^ Adam^42^ was used as the optimizer for stochastic gradient descent, and the minibatch size was set to 32. Training of CNN was carried out in 50 epochs using training data, and performance against test data was evaluated using CNN when classification accuracy for validation data became maximal. CNN training was performed 5 times with different random seed, and the mean and standard deviation were reported.

We also implemented the focus classification model using logistic regression based on a previous study^27^. In the feature extraction process, a focus candidate image is binarized using the saturation value in the HSV color space and the threshold value determined by the discriminant analysis method. We regarded the white pixels as the focus region. Then, three hand-crafted features, median (MD), equivalent diameter (ED), and weighted perimeter difference (WPD), were extracted from the segmented region. MD is the median value of the grayscale pixel value of the focus region. ED is the diameter of a circle whose area is equal to the focus region. WPD was calculated using the following equation:

$$\left( {{\text{FRP}} - {\text{EFP}}} \right) \times \left( {{\text{AREA}} - {\text{AREA}}_{{{\text{min}}}} } \right)/\left( {{\text{AREA}}_{{{\text{max}}}} - {\text{AREA}}_{{{\text{min}}}} } \right),$$

where ${\text{AREA}}_{{{\text{min}}}}$ and ${\text{AREA}}_{{{\text{max}}}}$ denote the minimum and maximum values of the focus area observed in the training data, respectively, and ${\text{AREA}}$ is the area of the focus region. ${\text{FRP}}$ and ${\text{EFP}}$ indicate the actual perimeter of the extracted focus region and perimeter of a circle whose area is equal to the focus region, respectively. A logistic regression model was trained to classify the focus candidate image from these three features. The same training and validation data used in the CNN training were used to train the logistic regression model.

### Conventional classification

All experimental procedures were performed in accordance with protocols approved by the institutional ethical committee, YNU Ethical Committee for Medical and Health Research (Authorization No. Hitoi-2021-06), following Ethical Guidelines for Medical and Health Research Involving Human Subjects from Ministry of Education, Culture, Sports, Science and Technology and Ministry of Health, Labour and Welfare, Japan. We obtained informed consent from all volunteers. Fifteen volunteers judged 281 focus-potential test data randomly selected from 1405 images. The test data is the same as the one for the CNN evaluation. Their age ranges from 21 to 36 years. They were beginners who have experience in cell culture but no experience in Bhas 42 CTA. They were briefed on the purpose of the Bhas 42 CTA, and learned the focus criteria and perspective of the image atlas using the OECD guidance document. Fifteen people performed positive / negative judgment for the same 281 test data. At this time, the time taken for the judgment was also measured. The evaluation was carried out using the accuracy rate and recall rate.

## Supplementary Information

Supplementary Information 1.

Supplementary Information 2.

Supplementary Information 3.
